# Children’s, parents’ and other stakeholders’ perspectives on early dietary self-management to delay disease progression of chronic disease in children: a protocol for a mixed studies systematic review with a narrative synthesis

**DOI:** 10.1186/s13643-017-0671-8

**Published:** 2018-01-25

**Authors:** Pearl Pugh, Pippa Hemingway, Martin Christian, Gina Higginbottom

**Affiliations:** 1 School of Health Sciences, University of Nottingham, Nottingham, UK; 2 Nottingham Children’s Hospital, Nottingham University Hospitals NHS Trust, Nottingham, UK

**Keywords:** Self-management, Chronic disease, Chronic kidney disease, Diet, Paediatric, Stakeholders, Disease progression, Perspectives

## Abstract

**Background:**

Chronic disease of childhood may be delayed by early dietary intervention. The purpose of this systematic review is to provide decision-makers with a perspective on the role of early dietary intervention, as a form of self-management, to delay disease progression in children with early chronic disease, as described by children, parents and other stakeholders.

**Methods:**

The study will systematically review empirical research (qualitative, quantitative and mixed method designs), including grey literature, using a narrative synthesis. A four-stage search process will be conducted involving a scoping search, the Scottish Intercollegiate Guidelines Network (SIGN) Patient Issues search filter on MEDLINE, the search of seven databases using a chronic disease and chronic kidney disease (CKD) search strategy, and hand searching the reference lists of identified papers for additional studies. All studies retrieved during the search process will undergo a screening and selection process against the inclusion/exclusion criteria. Methodological quality of relevant studies will be assessed using a validated Mixed Studies Review scoring system, before inclusion in the review. Relevant grey literature will be assessed for methodological quality and relative importance using McGrath et al.’s framework and the Academy Health advisory committee categories, respectively. Data extraction will be guided by the Centre for Review and Dissemination guidance and Popay et al.’s work. The narrative synthesis of the findings will use elements of Popay et al.’s methodology of narrative synthesis, applying recognised tools for each of the four elements: (1) developing a theory of how the intervention works, why and for whom; (2) developing a preliminary synthesis of findings of included studies; (3) exploring relationships in the data; and (4) assessing the robustness of the synthesis.

**Discussion:**

This mixed studies systematic review with a narrative synthesis seeks to elucidate the gaps in current knowledge and generate a fresh explanation of research findings on early dietary self-management in chronic disease, with particular application to CKD, from the stakeholders’ perspective. The review will provide an important platform to inform future research, identifying the facilitators and barriers to implementing early dietary interventions. Ultimately, the review will contribute vital information to inform future improvements in chronic disease. The lead author has a particular interest in CKD paediatric service delivery.

**Systematic review registration:**

The review has been registered with PROSPERO (CRD42017078130).

**Electronic supplementary material:**

The online version of this article (10.1186/s13643-017-0671-8) contains supplementary material, which is available to authorized users.

## Background

Chronic diseases or chronic illness, defined as a progressive, irreversible change to an individual’s health persisting for an extended period of time [1], consistent with having particular service needs [2], are the leading cause of morbidity and mortality worldwide [3]. An alarming 16–18% of children are now classified as having a chronic illness or a disease requiring significant ongoing health care needs [2, 4]. Chronic kidney disease, defined by the Kidney Disease Quality Outcome Initiative (K/DOQI) as any impairment to the kidney causing a decrease in the glomerular filtration rate (GFR) for 3 months or more [5], costs the National Health Service in the UK an estimated £1.44–1.45 billion annually [6], with much of the cost taken up in the provision of dialysis services that are needed when milder CKD progresses to established renal disease. In the UK each year, 60.4 per million age-related population under 16-year-olds are affected by CKD [7], and receiving dialysis significantly reduces a child’s life expectancy [7, 8]. Chronic kidney disease leads to raised phosphate which in turn is associated with vascular calcification and future cardiovascular morbidity and mortality [9, 10]. Raised phosphate also affects growth [8, 11]. Fibroblast growth factor-23 (FGF-23) is a bone-derived circulating peptide which has been shown to be associated with early development of CKD-Mineral Bone Disorder [11]. Fibroblast growth factor-23 has also been shown to be associated with CKD progression in children [12].

As shown in Table 1, CKD is classified by the estimated glomerular filtration rate (eGFR); it ranges from stage 1 to stage 5. During stages 2–3 CKD, abnormal biochemical changes in the bones begin, with a rise in FGF- 23 level, and a possible increase in plasma parathyroid hormone (PTH) levels in response to subclinical calcium and phosphate changes. Early indicators of CKD-Mineral Bone Disorder such as abnormal mineral metabolism, abnormalities of bone turnover and linear growth have been reported in almost 50% of children with CKD 3 [13].

**Table 1:**
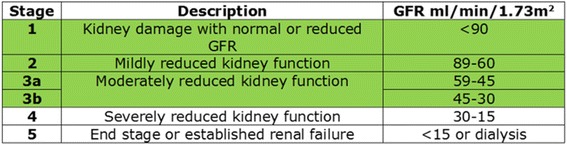
Stages of chronic kidney disease

The early stages of CKD are highlighted in green

In recent years, it has been shown that the provision of dietary phosphate lowering education as an early therapeutic strategy to lower phosphate uptake during the early stages of GFR decline may reduce fibroblast growth factor 23 levels and thus delay disease progression and the development of CKD-Mineral Bone Disorder [12–14]. Currently, however, it is a routine practice for specialist paediatric renal dietetic support to only be offered [15] when there is evidence of a rise in the serum phosphate level. As phosphate levels are normally within range until the GFR is less than 15–20 ml/min/1.73m^2^ [16]—CKD stages 4–5—failure to provide dietary intervention until this late stage may mean an important opportunity to slow CKD progression is being missed.

For children with a chronic illness, self-management is the interaction of health behaviours and related processes that children and families engage in to care for a chronic condition [17]. In many chronic diseases, early self-management principally involves dietary modification [18, 19]. Effective self-management cannot be undertaken in isolation but requires collaborative partnership with healthcare professionals [20]. Self-management is receiving increased attention in the adult CKD literature [18, 21, 22] and building momentum in the paediatric literature on chronic diseases [23–25]. However, there is a paucity of data on self-management in children with early CKD. Ultimately, self-management should provide a source of empowerment, improve health outcomes and provide useful coping strategies [18].

Prescribing a specialised diet in the early stages of a chronic disease may be met with resistance by the family. It may add to the ‘burden of care’ [26] and be disruptive to family life [27], all of which augments the mounting psychosocial pressures associated with the diagnosis of a chronic illness [27]. The asymptomatic nature of some chronic diseases [28] means that children do not visibly appear unwell, as the diagnosis is primarily based on biochemical markers. This creates a particular challenge for parents, necessitating the fostering of trusting relationships with healthcare professionals who will make principal decisions on the child and families behalf [29]. Healthcare professionals have a responsibility to offer the best treatment options to the patient and their families [30] as part of their professional code of ethics.

Therefore, given both the recognised need for early dietary phosphate intervention strategy as a form of self-management and the associated burden of care [26] on the family, the acceptability of an early dietary intervention strategy may be contingent on the patient, parental and other stakeholder perspectives as to its worth. Due to the paucity of data on early dietary self-management in children with early-stage CKD, this review will expand to focus on dietary interventions initiated during the early stages of any chronic disease in children; with a view to drawing lessons from other specialist disciplines to inform the practice of children’s early CKD management.

## Methods and design

### Study aim and objectives

The aim of this study is to provide decision-makers with perspectives on early dietary intervention, as a form of self-management, to delay disease progression in children with early chronic disease, as described by children, parents and other stakeholders. This will provide insight into the current thinking on early dietary self-management in children with chronic disease, and how this relates to early chronic kidney disease stages 1–3, in order to provide a strategy for service development. By its nature, the exploration of multiple stakeholder perspectives makes the focus of this review complex. A mixed studies approach, which appeals for a concomitant examination of qualitative, quantitative and mixed methods primary studies, will address the broad purpose of the scope, understanding and verification of knowledge grounded on all types of empirical research. A Mixed Studies Review with a narrative synthesis is a literature review approach in which the narrative element of qualitative, quantitative and mixed methods studies is systematically identified, selected, appraised and synthesised. Due to the complex and highly context-sensitive nature of interventions, a mixed studies review is particularly relevant to health science [31, 32]. A mixed studies review can provide a better understanding of a health issue than when one type of research approach is used alone. This mixed studies review will have an exploratory purpose where the qualitative component dominates. The use of Pluye et al.’s eight-stage Question Eligibility Source Identification Selection Appraisal Extract and Synthesis (QESISAES) framework [33], which is in line with the PRISMA statement (www.prisma-statement.org), will enable us to (1) to identify, appraise and synthesise qualitative, quantitative and mixed methods design empirical research; (2) to identify the facilitators and barriers to providing early dietary self-management for children with chronic disease and CKD 1–3; and (3) to disseminate the findings strategically via a managed paediatric renal network to influence commissioners and government level decisions. The review has been registered with PROSPERO (CRD42017078130).

### Research question

The PICO tool was used to define the research question (Table 2).

**Table 2:** PICO tool

Population—children aged 16 years or less, parents or carers, health care professionals involved in the provision of care and support of a child aged 0–16.
Intervention—dietary intervention as a form of self-management during the early stages of a chronic disease or CKD (stages 1–3).
Comparison—qualitative and mixed methods studies may not have a comparison group; quantitative studies may compare the given dietary intervention with usual care or standard practice.
Outcome—to delay disease progression.

The research question is as follows: What are the views of children, parents’ and others stakeholders’ towards the use of an early dietary intervention as a form of self-management, to delay disease progression, for children aged 0–16 years with an early chronic disease?

#### Knowledge gap on early dietary interventions for children with a chronic disease or CKD stages 1–3

Systematic evidence exists regarding carers and adult patient views on the needs and treatment decision-making of adults with a chronic disease [34, 35], and the experience of parents of children with chronic kidney disease [36]. However, a scoping review of databases (MEDLINE, CINAHL, Cochrane, EMBASE, AMED, Scopus and PsychoINFO, and the JBI Database of Systematic Reviews and Implementation Reports) revealed to date a mixed studies systematic review, systematic review or protocol is yet to be conducted or created on this topic.

### Inclusion criteria

#### Population or sample—types of participants

The review will consider studies that include one or more of the following: children aged 16 years or less; parents or carers, healthcare professionals but not exclusively the paediatrician, paediatric nephrologist, nurse or dietitian involved in the provision of care and support of a child aged 0–16 years that has early chronic disease or the early stages of chronic kidney disease (stages 1–3) in a hospital, home or community setting.

#### Phenomena of interest or intervention

The review will consider as the phenomena of interest the perspective and views of children, parents and other stakeholders of (1) dietary intervention as a form of self-management during the early stages of a chronic disease or CKD (stages 1–3); (2) how the intervention is delivered; (3) the challenges to providing early dietary intervention within a hospital, home or community setting.

#### Design—types of studies

The review will consider studies that use qualitative, quantitative or mixed methods research methods. The qualitative research studies are required to offer insight into the perspective of the multiple stakeholders. The quantitative studies will provide the context, with a focus on causality.

#### Evaluation—context or comparison

The review will include in-patient and out-patient hospital and community care, in any country, that record the perspectives and views of children, parents or other stakeholders towards early dietary self-management for children with early stages of a chronic disease or CKD (stages 1–3). Quantitative studies may compare the given dietary intervention with usual care or standard practice.

### Outcome

The outcome of the study is to delay the progression of the chronic disease.

#### Research type

The review will consider studies that focus on qualitative, quantitative or mixed methods research which has a narrative description, which is reported separately.

### Exclusion criteria

#### Sample—types of participants

The review will exclude studies with a primary diagnosis of acute kidney injury (AKI) or damage and where the chronic disease is caused by solid-organ tumours.

#### Phenomena of interest

The review will not consider as the phenomena of interest the perspective and views of children, parents and other stakeholders where (1) phosphate binder medication was used as the primary form of self-management.

Due to the linguistic abilities of the reviewers, only studies published in English will be included. This is an acknowledged limitation of the study.

### Search strategy

The search will utilise established systematic review methodologies as detailed in the Centre for Reviews and Dissemination Guide for Undertaking Reviews in Health Care [37] and integrate guidelines for the selection, appraisal and review of the grey literature [38, 39].

Health service researchers regularly produce and use grey literature to defend their inquiry. Chronic care and chronic care delivery are priority subject areas [39] for grey literature.

This Mixed Studies Review search strategy will cross multiple databases as it seeks to identify both published and unpublished studies using a four-stage approach. In order to retrieve maximum coverage of the literature, careful thought was given to the most appropriate databases to search. (1) An initial scoping search of MEDLINE, CINAHL, Cochrane, EMBASE, Scopus, PsycINFO and OpenGrey failed to identify sufficient literature on early dietary self-management in children with CKD. However, given the importance of diet in the management and prevention of progressive chronic disease, the findings suggested there would be sufficient literature to inform a systematic review if the search was extended to all chronic disease in childhood. (2) The Scottish Intercollegiate Guidelines Network (SIGN) (website www.sign.ac.uk) has a validated Patient Issues search filters; this was applied to MEDLINE. Despite providing a broad coverage of patient issues, it did not deliver the required specificity to address the research question. With the support of an information scientist (health research librarian), an analysis of the search terms and Medical Search Headings (MeSH) contained in the title and abstract was used to write a chronic disease and CKD search strategy to reflect the review question. (3) This search strategy was applied to seven databases (see Table 3). (4) Finally, the reference list of all identified papers will be hand searched for additional studies. Author searches and contact will be performed as necessary.

### Key search terms are anticipated to include among others

Chronic Disease OR Chronic Illness OR Chronic Disorder OR Chronic Kidney Disease OR Chronic Kidney Failure OR Renal Insufficiency OR CKD; AND Patient OR Adolescent OR Child; AND Parent OR Carer OR Nurse OR Consultant OR Physician OR Dietitian OR Allied Health Personnel; AND Self-management OR Self-care OR Early intervention OR Secondary prevention; AND Disease progression OR Time OR Endpoint; AND Diet OR Nutrition OR Diet therapy OR Food; AND Perspectives OR Views OR Concern OR Attitude.

A detailed search strategy for MEDLINE is shown in Additional file 1.

### Screening and selection process

Using the criteria for inclusion (Table 4), relevant literature will be identified for further scrutiny.

**Table 3:** Database to be searched

Name	Provider
MEDLINE—In-Process & Non-Indexed Citations and Ovid Medline 1946	Ovid (1946 onwards)
EMBASE—Excerptra Medica Database	Elsevier (1986 onwards)
Cochrane Library	
CINAHL—Cumulative Index of Nursing and Allied Health Literature	EBSCO (1982 onwards)
PsycINFO	(1995 onwards)
Scopus	Elsevier (1970 onwards)
OpenGrey (System for Information on Grey Literature in Europe) www.opengrey.eu	INIST-CNRS (1997 onwards)

**Table 4:** Screening and selection criteria checklist

Criteria for inclusion	Yes	No	Cannot say
1. Involved children (≤ 16 years) with an early chronic disease or CKD (1–3)			
2. Reported children, parents’ or other stakeholders’ perspective towards dietary self-management			
3. Publication date January 2000 to the date of publication of the protocol			
4. Reported the inclusion of diet/nutrition/food or lifestyle changes as part of a self-management strategy			
5. Available in English			
6. Empirical research and findings

**Table 5:** Tools for narrative synthesis and risk of bias

Element	Tools for narrative synthesis and assessment of bias
2	Evans et al. (2002)—the synthesis process/tabulation A textual summary of the results in a tabular format with headings such as setting, participants, research aim, sampling technique, analysis and results.
2	Clarke and Braun (2006, 2013)—thematic analysis The data presented in tabular format will allow papers to be grouped according to themes that appear between and within studies. Themes may relate to particular features such as dietary intervention, stakeholder groups or main findings. Recurrent themes will be analysed using thematic analysis.
3	Popay et al. (2006)—Narrative synthesis/investigate heterogeneity/conceptual mapping Identify and investigate key characteristics that vary between the studies in order to elucidate the different findings. Clinkenbeard (1991)—Idea map or spider diagram Explore connections reported across the studies.
4	Popay et al. (2006)—Robustness of the synthesis Lincoln and Guba (1985) [52]—Assessment of bias Critical reflection will focus on (1) identifying study limitations and its impact on the findings; (2) risk of bias (quality, validity and generalisability (for quantitative research) and a focus on confirmability, transferability, credibility and trustworthiness (for qualitative research); (3) differences and uncertainties in the findings and how addressed; (4) identify aspects of the research where the evidence is weak or absent; (5) suggestions for future research and lastly; (6) the evidence will be synthesised and presented to highlight the ‘thick’ and ‘thin’ findings, key differences and strengths.

**Table 6:** Example of data to be extracted

The type of data to be extracted may include the following	
General study information	First author, publication year, title, contact author, publication source, country of the main author.
Characteristics of the methods	Country of study, the age of the group in receipt of self-management, the study design, participants and more specifically the type of stakeholder offering a perspective, number of participants, study duration, comparison group intervention, research method, data analysis and key findings.
Characteristics of any intervention	Identify the dietary intervention, how it was implemented, the resources used, who delivered the intervention, and any barriers and facilitators to implementing the intervention.
Perspective of stakeholders	Narrative detail describing stakeholder views, experience and perspectives towards a dietary intervention. This may be in the form of quotes or descriptive texted.

Studies will be imported into EndNote X7 reference manager. Duplicates will be excluded, and remaining studies will be subjected to a title, abstract and full-text sift against the inclusion/exclusion criteria. Studies that state lifestyle changes, without mention of a dietary component, will be excluded due to ambiguity.

**Fig. 1:**
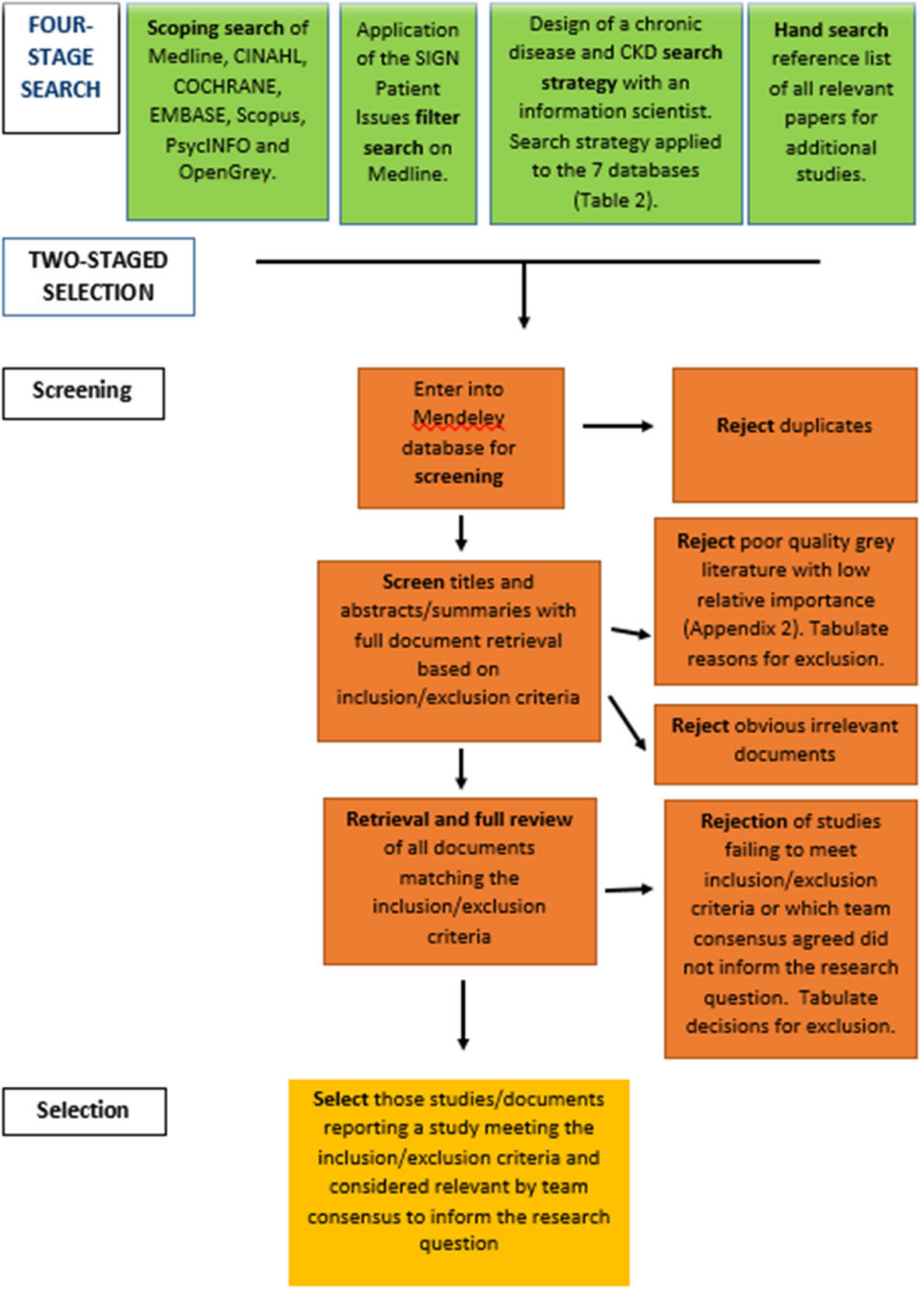
PRISMA (Preferred Reporting Items for Systematic Reviews and Meta-Analyses) flow chart sequencing the review process

All descriptive and analytic research study designs will be included [40]. Therefore, reviews, editorials, commentaries and expert opinion papers will be excluded, although the reference list of reviews will be searched for relevant literature. A PRISMA flow diagram will present the results of the search process [41] (Fig. 1).

### Appraisal of the methodological quality and relative importance of grey literature

Tyndall’s checklist (Additional file 2) will be used to appraise the quality and relative importance of grey literature [42]. The checklist will appraise the following aspect of grey literature: authority, accuracy, coverage, objectivity, date and significance.

Grey literature that passes the appraisal must present a clear research question(s), with key findings, and give adequate details on the population studied, interventions, and study design, a method of analysis and evaluation outcomes [38].

### Assessment of methodological quality

All relevant studies retrieved through the search strategy will be appraised for methodological quality by a primary and secondary reviewer before inclusion in the study. The Mixed Methods Appraisal Tool (MMAT) [43, 44] and scoring system (Additional file 3) will be used. It is an efficient and validated tool [45] (Additional file 4), and the only available tool that allows the concomitant quality appraisal of qualitative, quantitative and mixed methods studies (mixed studies reviews). Any disagreements that arise between the reviewers will be resolved through discussion, achieving consensus with a third reviewer if necessary.

### Data extraction

A relevancy appraisal will be undertaken by the primary author by first reviewing the title and abstract. Potentially relevant articles which match the inclusion criteria will be retrieved, and the whole team (PP, PH, MC, GH) will decide on the final inclusion. Data extraction will primarily be undertaken by one researcher (PP); however, the whole team will meet to decide on which data variables to include in the data extraction checklist. The type of data to be extracted will be determined by the nature of the studies, with a focus on how best to answer the research question. The Centre for Review and Dissemination guidance [37] and Popay et al.’s work [46] provide a suggested focus for the data extraction. An example of the type of data that will be extracted is detailed in Table 5.

### Narrative synthesis

Narrative synthesis is a procedure for describing, comparing and combining heterogeneous qualitative findings and quantitative results using text and illustrations [47]. The synthesis will use elements of Popay et al.’s ([46], p. 5) methodology of narrative synthesis, which involves ‘telling a trustworthy story’. It is defined as an ‘approach to the systematic review and synthesis of findings from multiple studies that rely primarily on the use of words and text to summarise and explain the findings of the synthesis’ [46] (p. 5). Both qualitative, quantitative and mixed methods studies can utilise this approach, as the focus is on the interpretive synthesis of the narrative findings of the research as opposed to a meta-analysis of the data. This approach will provide access to the multi-disciplinary perspective of stakeholders and provide a dynamic viewpoint for evaluating the plurality of health-related knowledge on early dietary self-management in chronic diseases, using multiple methodologies.

A narrative synthesis comprises four key elements:

1. Developing a theory of how the intervention works, why and for whom
2. Developing a preliminary synthesis of findings of included studies
3. Exploring relationships in the data
4. Assessing the robustness of the synthesis

We will follow the steps outlined by Popay et al. [46].

The narrative synthesis brings on a cyclical process with an interweaving of the key elements. Within each element, various tools will be employed to suit the nature of the evidence. Many of these tools will assess the risk of bias.

#### Element 1: developing a theory

Theory development will not be undertaken because the review is of an exploratory nature.

#### Element 2: developing a preliminary synthesis

The preliminary synthesis will consist of extracting the descriptive characteristics of the studies in a table, producing a textual summary of the results. The tabulation process will help to develop an initial description of the included studies and begin to identify patterns across studies. The table will likely provide details on the study design, results of study quality assessment, outcome measures and other results. Tabulation will provide a descriptive synthesis of data, allowing the researcher to review and compare results between studies and to express their own views of the body of research. Additionally, differences in study populations, methods of data collection and data analysis will be easier to identify. Tabulation will provide a list of the studies’ characteristics and therefore will provide an important building block for undertaking the next elements of the synthesis process [47]. Thematic analysis will then be used to allow key themes to emerge from the studies [48, 49] (Additional file 5). To assist this process, we will use the data analysis software ATLAS.ti (http://atlasti.com/). The author PP will receive instruction from GH, a registered ATLAS.ti trainer.

#### Element 3: exploring relationships within and between studies

Exploration of relationships within and between studies will highlight factors facilitating the impact of an intervention, or explanations of how or why a component has a particular impact. Patterns of study characteristics and reported findings emerging from the studies will be subject to rigorous evaluation to identify factors that may explain differences in stakeholders’ perspectives, revealing any facilitators and barriers to implementing early dietary self-management. These patterns will be evaluated alongside key aspects reported in other literature. Careful attention will be paid to the heterogeneity, which is the clinical variation in outcomes of research methods, methodologies and participant characteristics, interventions and other unknown sources across the studies, using narrative synthesis methods. Narrative methods are a valuable tool for investigating heterogeneity across primary studies, highlighting components of an intervention which may account for its success or investigating the possibility that study variation is due to theoretical variables [46].

#### Conceptual mapping

After interrogating the literature using thematic analysis and examination of heterogeneity, a conceptual model will be drafted to provide a visual representation of the state of knowledge about the different stakeholder perspectives on early dietary self-management in chronic disease. This will help to highlight the facilitators and barriers to implementation and identify areas that require further research. Several drafts will be made, comparing and contrasting the multiple stakeholders’ views, before arriving at the final conceptual map [46]. The model may also take the form of an idea map or spider diagram [50].

#### Element 4: assessing the robustness of the synthesis

The conclusion will include a critical reflection to assess the robustness of the synthesis process. This will involve an assessment of the strength of the evidence for drawing conclusions about the stakeholder’s perspectives and an assessment of the transferability of the synthesis findings to different population groups or contexts. The key to making certain the robustness of the synthesis is the methodological quality of the included studies and the analytical methods employed to develop the narrative synthesis. The summary discussion, as recommended by Popay et al. [46], will address the factors listed in Table 6.

### Dissemination of the findings and recommendations

It is our aim to apply the findings of the review to the management of CKD in childhood, with the anticipation that other relevant paediatric specialities will do likewise. The main findings of the review will be disseminated to key stakeholders across a managed paediatric nephrology network, in order to impact policy and practice change in early paediatric nephrology service delivery. The managed network has an online resource hub for professionals, children and carers which would serve as a powerful dissemination vehicle. Likewise, the network holds bi-annual multi-professional team education and training meetings where the findings can be publicised. Publication of the findings in accessible health science journals will make a contribution to academic theory and practice. Also, as the lead author is engaged in clinical practice with children with early CKD and their families, face to face dissemination will be ongoing.

## Discussion

This mixed studies systematic review with a narrative synthesis seeks to elucidate the gaps in current knowledge and generate a fresh explanation of research findings on early dietary self-management in chronic disease, from the stakeholders’ perspective. The review will provide an important platform to inform future research, identifying the facilitators and barriers to implementing early dietary interventions. Ultimately, the review will contribute vital information to inform future improvements in CKD paediatric service delivery, with an impact on healthcare utilisation and costs.

## Additional files

Additional file 1: Detailed search strategy example on MEDLINE. (DOCX 13 kb)

Additional file 2: The AACODS checklist for the evaluation and critical appraisal of grey literature. (DOCX 585 kb)

Additional file 3: A scoring system for mixed studies reviews [51]. (DOCX 307 kb)

Additional file 4: Mixed Methods Appraisal Tool. (DOCX 520 kb)

Additional file 5: Six phases of thematic analysis (Braun & Clarke, 2006). (DOCX 449 kb)

## Abbreviations

CKD: Chronic kidney disease
eGFR: Estimated glomerular filtration rate
FGF-23: Fibroblast growth factor-23
GFR: Glomerular filtration rate
K/DOQI: Kidney Disease Quality Outcome Initiative
MeSH: Medical Search Headings
MMAT: Mixed Methods Appraisal Tool
PRISMA: Preferred Reporting Items for Systematic Reviews and Meta-Analyses
PTH: Parathyroid hormone
QESISAES: Question Eligibility Source Selection Appraisal Extract and Synthesis
SIGN: Scottish Intercollegiate Guidelines Network
