# Supplementary material for: Children’s, parents’ and other stakeholders’ perspectives on early dietary self-management to delay disease progression of chronic disease in children: a protocol for a mixed studies systematic review with a narrative synthesis
Source: Syst Rev. 2018 Jan 25;7:20. doi: 10.1186/s13643-017-0671-8 (PMC5785819; doi:10.1186/s13643-017-0671-8)
Supplement: Supplementary file 1 — Detailed search strategy example on MEDLINE. (DOCX 13 kb) [file 13643_2017_671_MOESM1_ESM.docx]

# Additional file 1. Detailed search strategy example on Medline

| Identifiers I (Subject headings that relate to the term ‘Chronic Disease’ **OR** ‘Chronic Kidney Disease’):   - Chronic Disease - Chronic Illness - Chronicadj2(illness* or disease* or disorder*)   **‘OR’**   - Renal Insufficiency - Chronic Kidney Failure - Kidney Disease   **‘AND’**  Identifier II (Subject headings that relate to the term ‘child’ combined with **‘OR’:**   - Child, Preschool - Infant - P*ediatric - Adolescent |
| --- |
| **‘AND’** |
| Identifiers III(Subject headings that relate to the term ‘Early Intervention’ combined with **‘OR’:**   - Secondary prevention - Early intervention (education) - Self-care - Self-management (keyword) |
| **‘AND’** |
| Identifiers IV (Subject headings that relate to the term ‘stakeholder’ combined with **‘OR’:**   - Patients - Parents - Caregivers - Nurses - Physicians - Consultants - Nutritionist - Allied Health Personnel |
| **‘AND’** |
| Identifier V (subject headings that relate to the term ‘diet’ combined with **‘OR’:**   - Nutrition therapy - Diet* therapy - Diet - Dietary (keyword) - Food |
| **‘AND’** |
| Identifier VI (Subject headings that relate to the term ‘disease progression’ combined with **‘OR’:**   - Time - Time factors - Disease progress (keyword) - Endpoint determination |
| **‘AND’** |
| Phenomena (Subject headings that relate to the term ‘perspectives’ combined with **‘OR’:**   - Attitude - Attitude to health - Value of life - Knowledge - Perception - Emotions - Health knowledge, attitude, practice - Health behaviour |
