# Supplementary material for: Children’s, parents’ and other stakeholders’ perspectives on early dietary self-management to delay disease progression of chronic disease in children: a protocol for a mixed studies systematic review with a narrative synthesis
Source: Syst Rev. 2018 Jan 25;7:20. doi: 10.1186/s13643-017-0671-8 (PMC5785819; doi:10.1186/s13643-017-0671-8)

# Additional file 2. The AACODS checklist for the evaluation and critical appraisal of grey literature


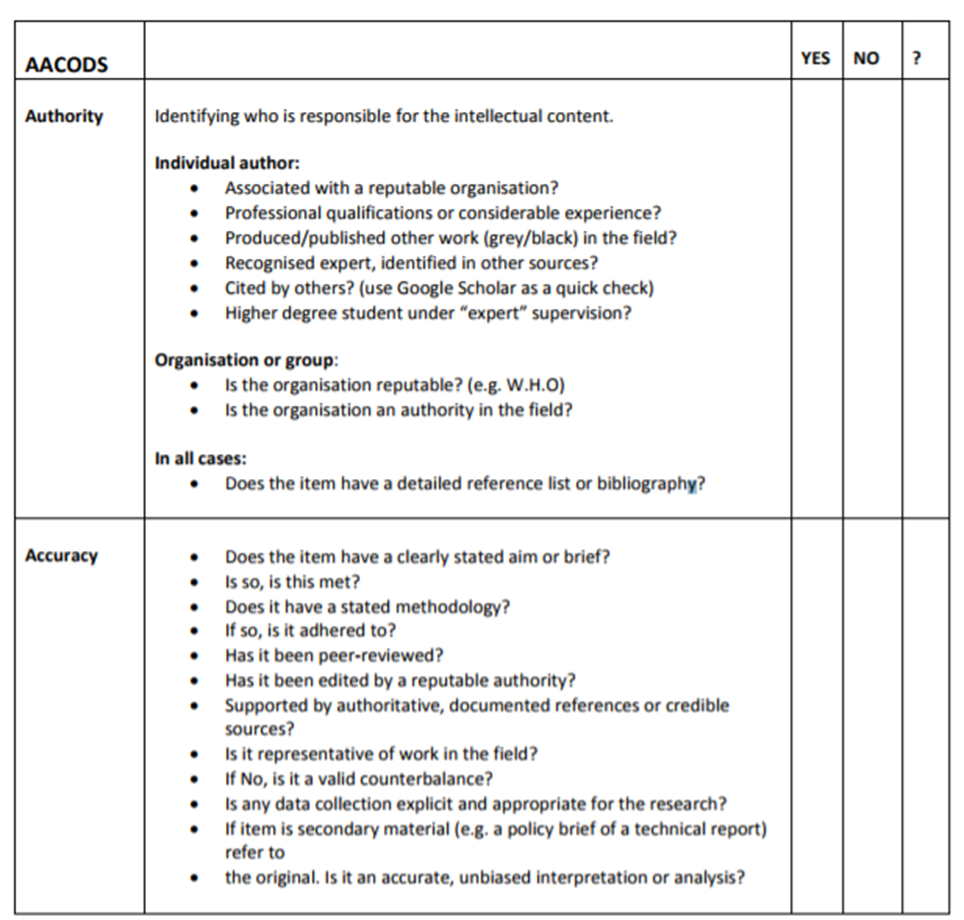


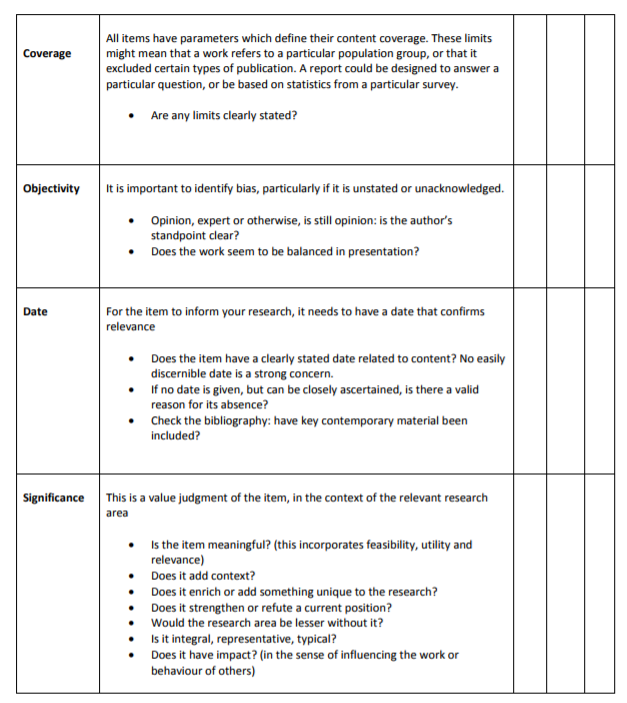

Supplement: Supplementary file 2 — The AACODS checklist for the evaluation and critical appraisal of grey literature. (DOCX 585 kb) [file 13643_2017_671_MOESM2_ESM.docx]
