# Supplementary figures and images for: Children’s, parents’ and other stakeholders’ perspectives on early dietary self-management to delay disease progression of chronic disease in children: a protocol for a mixed studies systematic review with a narrative synthesis
Source: Syst Rev. 2018 Jan 25;7:20. doi: 10.1186/s13643-017-0671-8 (PMC5785819; doi:10.1186/s13643-017-0671-8)

# Additional file 3. A scoring system for mixed studies reviews


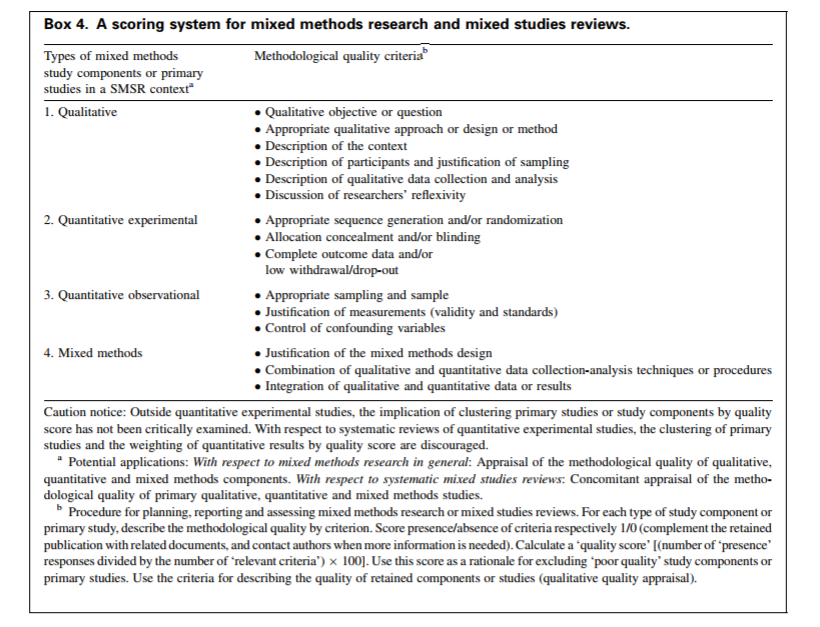

Supplement: Supplementary file 3 — A scoring system for mixed studies reviews [51]. (DOCX 307 kb) [file 13643_2017_671_MOESM3_ESM.docx]

# Additional file 4. Mixed Methods Appraisal Tool


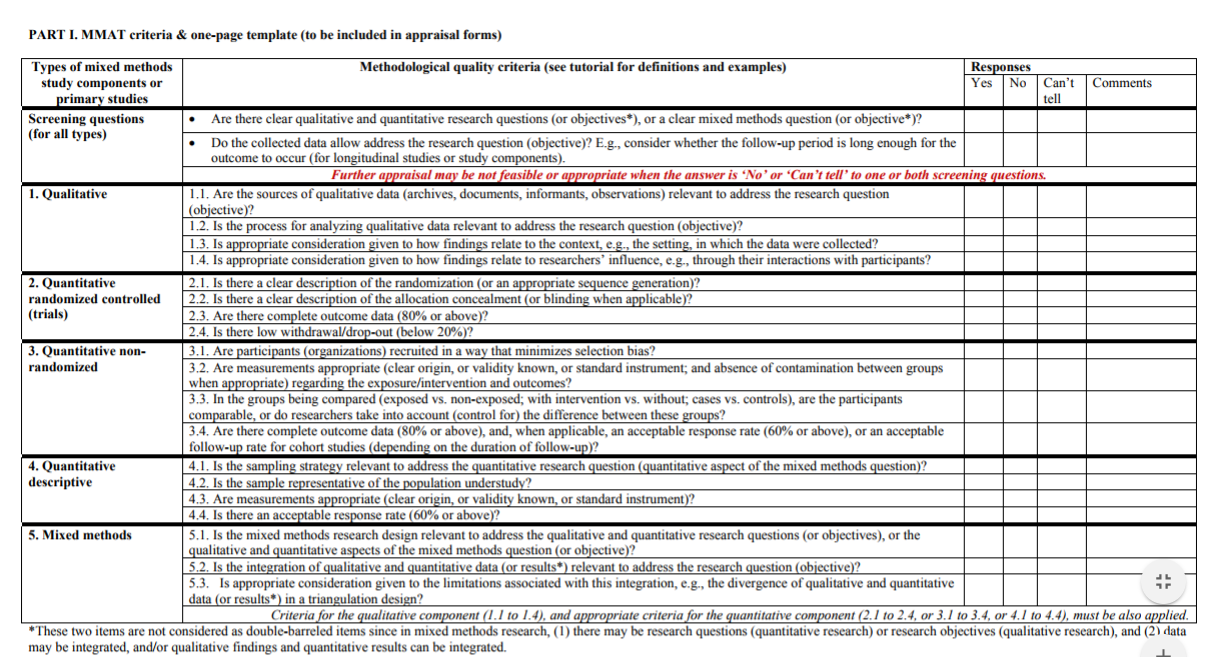

Supplement: Supplementary file 4 — Mixed Methods Appraisal Tool. (DOCX 520 kb) [file 13643_2017_671_MOESM4_ESM.docx]

# Additional file 5. Six phases of thematic analysis (Braun & Clarke, 2006)
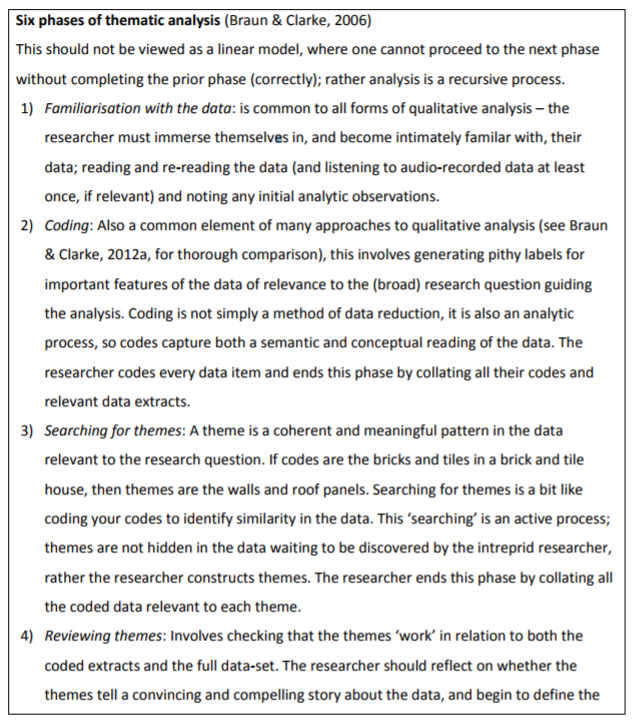


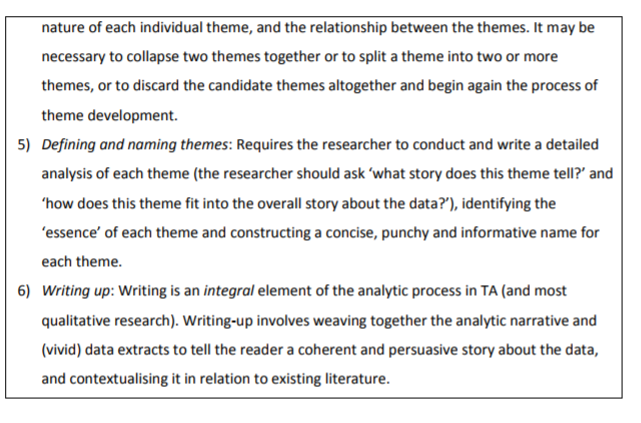

Supplement: Supplementary file 5 — Six phases of thematic analysis (Braun & Clarke, 2006). (DOCX 449 kb) [file 13643_2017_671_MOESM5_ESM.docx]
